# Incidence of traumatic dental injuries associated with orotracheal intubation in general anesthesia in children during mixed dentition in Damascus, Syria: a prospective longitudinal study

**DOI:** 10.1038/s41405-024-00276-7

**Published:** 2024-11-27

**Authors:** Mohammed N. Al-Shiekh, Mohamed Altinawi, Bana Darwish, Mawia Karkoutly

**Affiliations:** Department of Pediatric Dentistry, Faculty of Dentistry, Damascus University, Damascus, Syrian Arab Republic

**Keywords:** Oral diseases, Dental trauma

## Abstract

**Objectives:**

Endotracheal tube intubation by laryngoscope during general anesthesia is a safe procedure with a few complications. However, it may cause some damage to the oral cavity structures, which leads to postoperative pain and discomfort. Traumatic dental injuries associated with endotracheal tube intubation are one of the most common complications. The study aimed to determine the incidence of traumatic dental injuries during oral-endotracheal tube intubation in general anesthesia among children receiving surgery at the Children’s Hospital in Damascus City during 2022–2023.

**Methods:**

It was a prospective longitudinal study which investigated the incidence of traumatic dental injuries during oral-endotracheal tube intubation under general anesthesia in 110 children aged 6–12 years old at the University Children’s Hospital in Damascus City in 2022. After ensuring that the child met inclusion criteria and obtained written consent. Each child was examined before, during, and after 12–24 h of entering the operation room. Personal information (gender – age), information related to anesthesia procedures, and some oral cavity characters were collected.

**Results:**

The incidence of traumatic dental injuries during general anesthesia was 9.1%. Most of them intra-oral soft tissue injuries. The concussion is the most common injury related to teeth damage. In addition, the tongue was the most common-place. There is a correlation between the incidence of traumatic dental injuries and the difficulty of intubation, the number of intubation attempts, Mallampati score (*p* < 0.05).

**Conclusion:**

The anesthesiologist should evaluate the condition of each patient carefully. Document every detail in their record and inform the patient of the possibility of dental damage during the procedures especially in the case that has difficulty intubation.

## Introduction

Traumatic Dental Injuries (TDIs) are one of the most widespread public health issues that negatively affect emotional, psychological, physical, and financial aspects and quality of life [1]. The literature confirms a high worldwide prevalence of TDIs with broader geographic variations where TDIs affect almost 25% of school-age children and 33% of adults [2, 3]. Most injuries happen before the age of 19 [2, 4, 5]. TDIs are attributed to a diverse and complex set of factors [2, 3, 6]. Glendor et al. [7] suggested that the risk factors associated with TDIs can be divided into three main categories, including oral factors, such as increased overjet [8], environmental factors, including material deprivation [9], and human behavior like sport [9], child abuse [10], and obesity [11]. However, although this classification is beneficial, it is not comprehensive. Some risk factors cannot be included within the three categories mentioned above, such as gender, presence of disease, and learning difficulties [3]. One of the risk factors that correlate with TDIs is iatrogenic injuries (II). These injuries arise from iatrogenic conditions caused by doctors or dentists while providing treatment [6]. Endotracheal tube Intubation (ETI) during General Anesthesia (GA) or emergency intubation is one of the most common procedures associated with TDIs [12–14]. Furthermore, TDIs during GA are the most common cause of malpractice complaints against anesthesiologists [15]. Airway damage occurs commonly during both laryngoscopy and intubation [16].

The incidence of TDIs during GA ranges between 0.03% [17] and 1.13% [18] in retrospective studies and may reach 38% [19] if the studies are prospective and performed by a professional dentist [20, 21]. Poor oral hygiene [22, 23], aggressive intubation due to difficulty viewing the epiglottis [24], inadequate anesthesia [25], emergency intubation [15], lack of alternative intubation equipment [22], shortage in using protective measures [15], and anatomical difficulties [18] are some of the risk factors associated with TDIs during GA. It is accepted practice to conduct an oral examination before an operation, record the findings, and inform the patient of the incidence of TDIs. Also, In the event of dental injury, an anesthesiologist must be capable of immediate and proper management [26]. However, Ansari et al. [27] stated that TDIs associated with ETI during GA are not widely studied in the medical literature. In addition, Windsor et al. [28] mentioned that the most common age susceptible to TDIs during GA is Mixed Dentition (MD). Therefore, the study aimed to determine the incidence of TDIs during Oral-ETI in GA among children receiving surgery at the Children’s Hospital in Damascus City during 2022–2023.

## Materials and methods

### Study design and ethics

It is a longitudinal, descriptive, prospective study that investigates the incidence of TDIs during Oral-ETI by traditional laryngoscope in GA in children aged 6–13 years old and who received elective surgery at the Children’s Hospital in Damascus between December 2022 and December 2023. All procedures were carried out in conformity with all current ligations and guidelines. In addition, ethical approval was obtained from the Scientific Research Committee of the Damascus University, Syria (IRB No. UDDS-2599-09052022/SRC-1550). The study was carried out in adherence to STROBE checklists. Before data collection, all participants’ legal guardians provided informed consent.

### Sample size

The sample size was calculated according to the Cochran formulation [29]: n_0_ = Z^4^.p.q/e^4^.

According to the Nahas et al. [30] study, which set a confidence level of 95.5% with z = 2, e = 10%, and *p* = 50%, the minimum required sample size was 100, giving a power of 90%.

However, the sample size was increased to 110 to increase the level of accuracy and validity. The anticipated number of subjects who will drop out of the study was taken into consideration when increasing the sample size.

### Participants’ selection

This study included children aged 6–12 in mixed dentition who received surgical procedures under GA. All uncooperative patients, according to Frankl’s scale, were excluded. Children who received maxillofacial and upper respiratory tract surgery and those whose guardians refused to participate were excluded. In addition, the children who had cognitive and mental problems that banned them from active verbal connection and who had orthodontic appliances were excluded.

### Variables and data collection

After ensuring that the child met the inclusion criteria and obtaining written consent, each child was examined clinically separately before entering the operation room. The patient was examined during the operations, during the awake phase, and 12–24 h after the end of the intervention. During GA, they will undergo Oral-ETI utilizing a Macintosh blade (Welch Allyn Standard Laryngoscope Blade- English MacIntosh- Size 2. MFID: 69242, New-Med Instruments, Sialkot, Pakistan).

The following data was collected: demographic information (age - gender) - information related to aspects of anesthesia procedures (year of the residents were responsible for anesthesia - the resident’s assessment of the difficulty of intubation - number of intubation attempts - duration of anesthesia) - information related to the oral cavity and the anatomical structure (evaluation of the temporomandibular Joint – inter-incisor distance opening - the distance between the mental and the thyroid cartilage - occlusal classification of inter-molar relationship - Mallampati score – DMFT+dmft index - gingivitis).

In the event of TDIs, the diagnostic protocol found by Jones et al. [31] was used with some modifications, which consisted of Extraoral examination (facial fractures - wounds - bruises - swelling - abrasions - foreign bodies - deviation of the temporomandibular joint – bleeding). Intraoral examination (examination of the oral mucosa: upper lip, lower lip, frenulum, vestibular mucosa, gingiva, uvula, tongue, the floor of the mouth - Examination of the teeth and their identification according to the form of the injury: (avulsion - lateral luxation - intrusion - concussion - Subluxation - cracks - coronal fractures - root coronal fractures - pulp exposure - movement - percussion).

### Statistical methods

The Statistical Package for Social Sciences (SPSS)(IBM Corp., Armonk, NY, USA) was used for all analyses. The *p*-value was considered significant if it was less than 0.05. Descriptive statistics was used to describe the frequency and percentage of categorical variables and the mean, standard deviation, minimum, and maximum for numeric data. After Kolmogrov–Smironov test was used. The Mann–Whitney U test was used to compare numeric variables (age - number of intubation attempts - duration of anesthesia – inter-incisor distance opening - the distance between the chin and the thyroid cartilage - DMFT+dmft index) according to TDIs occurrence. In addition, the Chi-square test was used to compare categorical variables and their correlation to TDI incidence.

## Results

A total of 110 children (64.5% male and 35.5% female) were included in this study, the majority of them (92.7%) have a normal TMJ and more than half (56.4%) have a class I inter-molar relationship. However, most of them (81.8%) had no sign of gingivitis (Table 1). Regarding the years of residents, who were responsible for intubation, over a third were in the second year, and 80.9% estimated the intubation procedure wasn’t difficult (Table 1). Moreover, the mean age of participants in this study was 8.64 ± 1.86. In addition, the minimum number of intubation trials was one attempt while the maximum was 9 attempts. The mean duration of anesthesia was 2.5 ± 1.12 h. In addition, the mean inter-incisor distance was 3.5 ± 0.5 cm. Finally, the mean DMFT+dmft index for the children’s sample was 6.07 ± 4.01 (Table 2). Indeed, 9.1% of the individuals sample suffered from TDIs during ETI in GA. 80% intraoral and 20% extraoral (Table 3). All of those injuries were single, not multiple. All the extraoral TDIs were related to the temporomandibular joint. Where the intraoral TDIs were distributed as follows: 62.5% oral mucosa (60% tongue, 40% upper lip) and 37.5% related to teeth (66.7% concussion, 33.3% avulsion) (Table 3). Concussion injury occurred on teeth 11 while just tooth 51 was exposed to an avulsion injury. *The chi-square test* showed that those who were classified as difficult to intubate had a higher chance of suffering from TDIs. In addition, those who had a III or IV Mallampati classification, class II inter-molar relationship, and gingivitis presence had a higher opportunity to occur TDIs during ETI in GA (*p* < 0.05) (Table 4). It should be noted that *the Man–Whitney test* showed that there wasn’t a significant difference between the two groups associated with the age of the child, duration of anesthesia, inter-incisor distance, the distance between chin and thyroid cartilage, and DMFT+dmft index (*p* > 0.05) (Table 5).

**Table 1:** How categorical variables were distributed within the sample.

Categorical variables		*N*	%
Sex	Male	71	64.5
	Female	39	35.5
Year of the residents	1	19	17.3
	2	36	32.7
	3	29	26.4
	4	26	23.6
The resident’s assessment of the difficulty of intubation	It was difficult	21	19.1
	It wasn’t difficult	89	80.9
Evaluation of the Temporomandibular Joint	Normal	102	92.7
	Click on both sides with limited open-mouth	6	5.5
	Click on the left-side	2	1.8
Occlusal classification of inter-molar relationship	Class I	62	56.4
	Class II	16	14.5
	Class III	4	3.6
	Straight line	2	1.8
	Mesial step	21	19.1
	Distal step	5	4.5
Mallampati score	I	43	39.1
	II	38	34.5
	III	12	10.9
	IV	17	15.5
Gingivitis	Yes	20	18.2
	No	90	81.8

*N* number of children, % percentage of children.

**Table 2:** Statistical indicators of the sample.

	Minimum	Maximum	Mean	SD^a^
Age	6.00	12.00	8.6455	1.86388
Number of intubation attempts	1.00	9.00	2.8909	1.95990
Duration of anesthesia	0.50	6.00	2.5918	1.12528
Inter-incisor distance	2.30	4.80	3.5645	0.56821
The distance between the mental and thyroid cartilage	4.50	9.50	7.0973	1.20141
DMFT+ dmft index	0.00	18.00	6.0727	4.10797

^a^Standard deviation.

**Table 3:** Distribution of Traumatic dental injuries during oral-EndoTracheal Intubation in General Anesthesia.

		*N*	% of TDIs	% of the total sample
Incidence of TDIs during ETI in GA		10		9.1
Place of TDIs	Intra-oral	8	80	7.27
	Extra-oral	2	20	1.82
Extra-oral TDIs	Pain in TMJ	1	50	0.91
	Pain in TMJ with the limited opening of the mouth	1	50	0.91
Intra-oral TDIs	Soft tissue	5	62.5	4.54
	Hard tissue	3	37.5	2.72
Type of hard tissue TDIs	Concussion	2	66.7	1.82
	Avulsion	1	33.3	0.91
Location of soft tissue TDIs	Tongue	3	60	2.72
	Upper lip	2	40	1.82

**Table 4:** Chi-square test to compare between categorical variables according to incidence of TDIs.

		Incidence of TDIs during ETI in GA				*p*-value
		No		Yes		
		*N*	%	*N*	%	
Sex	Male	62	87.4	9	12.6	0.094
	Female	38	97.5	1	2.5	
year of the residents	1	18	94.8	1	5.2	0.411
	2	32	88.9	4	11.1	
	3	26	89.7	3	10.3	
	4	24	93.4	2	7.6	
the resident’s assessment of the difficulty of intubation	Difficult	14	66.7	7	33.3	0.000299^a^
	Not difficult	86	96.63	3	3.37	
evaluation of the Temporomandibular Joint	Normal	94	92.2	8	7.8	0.097
	Click on both sides with limited open-mouth	4	66.7	2	33.3	
	Click on the left-side	2	100	0	0.0	
occlusal classification of inter-molar relationship	Class I	60	96.8	2	3.2	0.000083^a^
	Class II	10	63.5	6	37.5	
	Class III	2	50	2	50	
	Straight line	2	100	0	0.0	
	Mesial step	21	100	0	0.0	
	Distal step	5	100	0	0.0	
Mallampati score	I	43	100	0	0.0	0.0061^a^
	II	35	92.1	3	7.9	
	II	9	75	3	25	
	IV	13	76.5	4	23.5	
Gingivitis	Yes	15	75	5	25	0.017^a^
	No	85	94.4	5	5.6	

*N* number of cases, *%* percentage of cases according to incidence of TDIs.

^a^Significant difference *p*-value < 0.05.

**Table 5:** Man–Whitney test to compare between numerical variables according to the incidence of TDIs.

	Incidence of TDIs		*p*-value
	No	Yes	
	Mean	Mean	
Age	8.54	9.7	0.098
Number of attempts	2.69	4.9	0.00013^a^
Duration of anesthesia	2.6	2.5	0.96
Inter-incisor distance	3.58	3.4	0.38
The distance between the mental and thyroid cartilage	7.16	6.47	0.078
DMFT+dmft index	23.26	23.6	0.54

^a^A significant difference.

## Discussion

This study investigated the incidence of TDIs during oral ETI in GA among children aged 6–13 who received elective surgery in a Children’s Hospital in Damascus between 2022 and 2023. For this purpose, 110 children were recruited, and it was found that the incidence of TDIs was 9.1%.

This percentage is higher than the percentages mentioned in retrospective studies. Newland et al. study [22] 0.04%, Kakei et al. study [32] 0.06%, Kuo et al. study [33] 0.05%, Martin et al. study [34] 0.2%, and Vogel et al. study [18] 1.13%. However, this value is less than what was found in Christensen et al. study [35] 22%, Mourão et al. study [36] 25%, and Manifar et al. study [37] 25%. If the research is conducted by a skilled dentist and is prospective [20, 21], the percentage could potentially climb to 38% [19]. This variance may attributed to differences in study design and sample size in addition to the age of participants and the character of the sample community. Oral mucosa injuries were higher than hard tissue injuries. This result is consistent with what Manifar et al. study [37] found and explained by the type of equipment used during laryngoscopy and intubation which makes easier to it harm soft tissue than hard tissue [14]. The most common type of injury related to hard tissue is Concussion. This result contrasts many studies [18, 19, 23, 35, 37] which found that uncomplicated crown fractures are the most common. In addition, one study found that avulsion is the most common [32]. This difference may be due to the absence of the percussion test from these studies, which is considered crucial in diagnosing clinically silent concussion injuries [38]. However, this study agrees with many studies [13–18, 22, 23, 25, 26, 28, 32, 35–43] published regarding the maxillary central incisor being the most affected teeth because of use it as a fulcrum when using a laryngoscope [14, 42]. A study mentioned that the right side is more vulnerable to injury compared to the left [22]. This result was found in this study and that can explained by the anthologists inserting the laryngoscope into the right side of the oral cavity initially, and when reaching the pharynx, turning the blade towards the left side [44]. In addition, this study found that the tongue was the most common location for TDIs in GA. This confirms what is mentioned in many studies [16, 19, 37].

There were 12.6% of male and 2.5% of female who had TDIs during GA. However, no significant difference was found related to gender and this is consistent with a group of studies [16, 19, 22, 32, 37]. This result can be interpreted by the surrounding circumstances environment of injured person play a more important role in the occurrence of TDIs comparing to factor like sex [2]. Moreover, no significant difference was resulted according to the age of children, this is supported by many studies [16, 17, 19, 37] and can be explained by the stage of age reflecting the same stage of occlusal development (Mixed dentition), which means similar features for all individuals’ sample. There wasn’t any real effect of the year of the residents on the occurrence of TDIs during ETI in GA. Gaiser & Castro study [45] mentioned the same result and that attributed to strict case evaluation criteria and continuous supervision by trained anesthesiologists.

Difficult intubation is a procedure that requires three or more attempts of intubation with a change in position, laryngoscope blade, or the use of a laryngeal mask [22]. This study found that 70% of TDIs in GA have difficult intubation and there was a significant difference between who had TDIs or not according to this variable when *Chi-square* was applied. However, many studies [16, 18, 36] don’t agree with this result. In addition, as the number of attempts to intubate increases the possibility of having TDIs increases. This fact confirms the result that was found when the difficulty of the intubation variable was studied. Mourão et al. Study [36] provide the same fact. Repeated intubation attempts require the anesthesiologist to apply a greater force (> 49 N) using the laryngoscope blade to see the epiglottis and this may lead to TDIs [24]. Most of the injuries happened to children who had a III and IV grade on the Mallampati score and there was a significant relationship between this variable and the incidence of TDIs during GA. This result is consistent with the Tan et al. study [46] and can be clarified by this score evaluating the oral soft tissues and the amount of space they occupy when the pharynx is visualized. The less the ability to visualize the pharynx (grade III-IV), the more difficult the intubation is and the more likely to damage the airway during intubation [47]. The mean of the DMFT+dmft index in children who had TDIs was 23.6 while in children who didn’t have TDIs was 23.26. However, there wasn’t any significant difference between the two groups (*p*-value > 0.05). This result doesn’t agree with what was mentioned in many studies [15, 17, 18, 22, 40] that can be explained by the fact that Syrian children have poor oral hygiene in two groups compared to the previous study’s participants [48]. Only a limited number of anesthesiologists know the importance and role of mouth guards during GA, laryngoscopy, and ETI procedures [20]. However, they play a fundamental role in protecting and managing joint pain on the one hand [49] and preventing or reducing the incidence of TDIs during intubation procedures on the other hand [50].

The main limitation of this study is the small number of participants. Longitudinal descriptive studies evaluate the incidence rate immediately after the injury. This type of study is considered expensive and time-consuming. It requires an integrated team to cover the largest possible number of injuries [51], unlike retrospective studies that depend on evaluating the prevalence rates by reviewing the data included by the anesthesiologists or by the patient if he realized the injury during a previous period. In addition, the age range in mixed dentition is limited, and it is difficult to follow up with children after general anesthesia. All of the above factors affected the sample size. In addition, this study does not evaluate other techniques in reducing dental traumatic injuries, such as miller blade, LMA Laryngeal mask airway, or video laryngoscope. Further research is needed to compare traditional laryngoscopes and alternative tools about the incidence of TDIs during oral-ETI in GA.

## Conclusion

The incidence of TDIs during oral-ETI in GA is 9.1% in children during mixed occlusal at Children’s Hospital in Damascus City. Even with the skilled hands of anesthesiologists, there is a chance of TDIs in GA. Moreover, higher intubation attempts, intubation difficulty, III or IV Mallampati score, class II inter-molar relationship, and gingivitis increased the possibility of injury during oral-ETI. The anesthesiologist should evaluate the condition of each patient carefully. Document every detail in their record and inform the patient of dental damage.

## Data Availability

The datasets generated during and/or analyzed during the current study are available from the corresponding author on reasonable request.
